# Site-specific evaluation of mutation-based mimics of histone glycation in the nucleosome

**DOI:** 10.3389/fmolb.2026.1838713

**Published:** 2026-05-18

**Authors:** Yazan Dalilah, Andreas Simm

**Affiliations:** University Clinic and Outpatient Clinic for Cardiac Surgery, Medical Faculty of the Martin Luther University Halle-Wittenberg, University Medicine Halle, Halle(Saale), Germany

**Keywords:** advanced glycation end product (AGEs), DNA breathing, histone glycation, histone-DNA interactions, molecular dynamics simulation (MD), mutation-based mimics, nucleosome, nucleosome dynamics

## Abstract

**Introduction:**

Histone glycation is a non-enzymatic post-translational modification (PTM) associated with aging and metabolic stress, yet its residue-specific structural and functional effects remain poorly understood. Because selectively installing defined glycation adducts is experimentally challenging, amino acid substitution by site-directed mutagenesis is commonly used to mimic such PTMs; however, the validity of these substitutions as models of specific glycation adducts has not been systematically assessed.

**Methods:**

Here, we performed atomistic molecular dynamics simulations of the nucleosome core particle to compare wild-type systems, advanced glycation end products (AGEs), and substitution-based mimics. Three sites were examined: H2BK43 and H4K31 modified as Nε-(carboxymethyl)lysine (CML), and H3R42 modified as methylglyoxal-derived hydroimidazolone (MG-H1), with glutamine used to mimic CML and tyrosine to mimic MG-H1.

**Results:**

Glutamine substitutions used to mimic CML reproduced the direction of local structural changes induced by CML at H2BK43 and H4K31, including increased protein contact, flexibility, and solvent exposure. In contrast, tyrosine substitution did not reproduce the effects of MG-H1 at H3R42, instead markedly reducing DNA engagement and electrostatic interactions. Microsecond-scale simulations further revealed a replicate-dependent propensity for asymmetric DNA entry/exit breathing in systems containing the H3R42Y mutation, a behavior not observed in wild-type or other modified systems.

**Discussion:**

These findings indicate that the reliability of mutation-based glycation mimics depends on both the specific glycation chemistry and the local structural context. Consequently, such models should be structurally or biochemically validated before being used to infer nucleosome dynamics or biological consequences.

## 1 Introduction

Eukaryotic DNA is compacted into chromatin, the fundamental structural unit of which is the nucleosome core particle (NCP). The NCP comprises ∼147 base pairs of DNA wrapped around a histone octamer containing two copies each of histones H2A, H2B, H3, and H4 (Luger et al., 1997; McGinty and Tan, 2015). Nucleosome stability is maintained by a fine-tuned network of electrostatic interactions and hydrogen bonds between histones and DNA. Perturbations at critical histone residues can therefore influence DNA wrapping, chromatin accessibility, and transcriptional regulation (Kornberg and Lorch, 1999; McGinty and Tan, 2015).

Histone post-translational modifications (PTMs) are central regulators of chromatin structure and genome function (Kornberg and Lorch, 1999; Bannister and Kouzarides, 2011). While enzymatic PTMs have been extensively characterized, non-enzymatic histone glycation has only recently emerged as a biologically relevant modification associated with aging, oxidative stress, and metabolic imbalance (Galligan et al., 2018; Zheng et al., 2019). Glycation generates advanced glycation end products (AGEs), among which CML and MG-H1 are particularly prevalent *in vivo* (Thornalley, 2008). These stable adducts alter residue charge, hydrogen-bonding capacity, and steric properties, directly modifying lysine and arginine residues that play key roles in histone–DNA interactions (Galligan et al., 2018; Zheng et al., 2019).

Despite increasing recognition of histone glycation as a contributor to chromatin dysregulation, its residue-specific structural consequences within intact nucleosomes remain insufficiently understood (Galligan et al., 2018; Zheng et al., 2019). Experimental investigation of individual glycation events is limited by the lack of robust models capable of selectively reproducing defined AGE modifications at specific residues.

Site-directed mutagenesis is widely used to mimic PTMs through amino acid substitutions with comparable physicochemical properties (Liang et al., 2023; Reda et al., 2024). However, to our knowledge, mutation-based mimics have not yet been systematically evaluated as models for histone glycation, and the structural validity of such approximations remains unclear, posing a significant challenge to accurately interpreting their biological implications. Given that even single-residue perturbations can propagate into global chromatin effects, validating such models is important before using them to infer site-specific biological effects.

**FIGURE 1:**
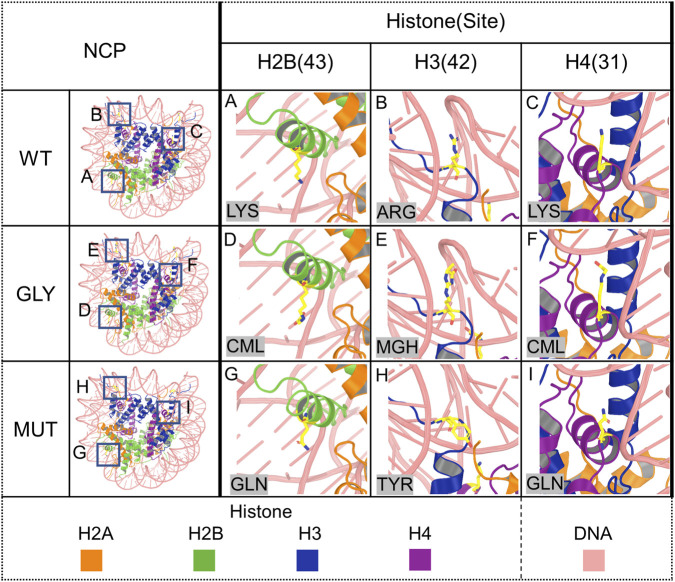
Structure of the nucleosome core particle (PDB ID: 5AV6) and close-up views of the analyzed histone sites and their glycation or mutation-based variants. Each change was introduced into both copies of the corresponding histone within the nucleosome; however, only one site is highlighted in yellow for clarity. **(A)** H2B K43. **(B)** H3 R42. **(C)** H4 K31. **(D)** H2B K43CML. **(E)** H3 R42MG-H1. **(F)** H4 K31CML. **(G)** H2B K43Q. **(H)** H3 R42Y. **(I)** H4 K31Q.

The present study provides an *in silico* assessment of mutation-based mimics for histone glycation at three biologically relevant sites identified in our laboratory: H2B-43CML, H3-42MG-H1, and H4-31CML. Glutamine (GLN) was examined as a proxy for lysine-derived CML, and tyrosine (TYR) as a proxy for arginine-derived MG-H1 (Figure 1). These substitutions were chosen to approximate some physicochemical consequences of glycation, rather than to reproduce the exact chemistry of the AGE adducts. GLN was selected because it is a neutral polar residue and was expected to approximate selected polarity and hydrogen-bonding features of the CML-modified side chain. TYR was selected as an empirical proxy for MG-H1 because its side chain provides a neutral, ring-containing polar functionality that was considered a simple way to approximate some features of the MG-H1 hydroimidazolone moiety, although it does not reproduce the exact chemistry of the adduct. Using atomistic molecular dynamics (MD) simulations, we compared wild-type, glycated, and mutation-substituted NCP systems to determine whether these substitutions reproduce glycation-induced changes in residue dynamics and local interactions.

The sites investigated here are subject to multiple PTMs, several of which have been linked to chromatin regulation and transcription (Casadio et al., 2013; Kim et al., 2013; Qin et al., 2019; Biggar et al., 2020; Millán-Zambrano et al., 2022). More specifically, H4K31 is a reported UFMylation target implicated in ataxia-telangiectasia mutated (ATM) activation, and mutation of this residue to Arg reduces ATM activation following ionizing radiation–induced DNA double-strand breaks (Qin et al., 2019). Histone H3R42 methylation has also been suggested to regulate transcription (Casadio et al., 2013). In addition, H3R42 lies within the ‘H3-latch’ (H3 residues 39–49), a region proposed to stabilize nucleosomal DNA ends (Armeev et al., 2021). On the other hand, H2BK43me2 was identified as a substrate of KDM5B and was found to undergo dynamic changes during stem cell differentiation (Biggar et al., 2020).

By integrating short (100 ns) simulations with extended microsecond trajectories, we distinguish local structural perturbations from longer-timescale consequences for nucleosome dynamics. This approach also allowed us to examine whether an unsuitable mutation-based mimic is associated with altered entry/exit DNA dynamics not observed for the chemically accurate glycation.

Together, this study provides a site-specific computational framework for evaluating mutation-based AGE models and underscores the importance of structural validation when interpreting the biological consequences of histone glycation.

## 2 Materials and methods

### 2.1 Preparing force field parameters for CML and MG-H1 modifications

Parametrization was performed for CML and MG-H1 as central fragments of an amino acid using the PyRED program (Bayly et al., 1993; Dupradeau et al., 2010; Vanquelef et al., 2011; Wang et al., 2013). The quantum-mechanics program interfaced by PyRED is firefly 8.2.0 QC package (Granovsky, n.d.), which is partially based on the GAMESS (US) (Schmidt et al., 1993) source code.

### 2.2 Building nucleosome systems for MD simulations

The nucleosome core particle (NCP) structure 5AV6 was used to build all systems. This NCP comprises the histone octamer wrapped by ∼147 bp of DNA, and lacks full-length histone N- and C-terminal tails. To prepare the systems, solvent and Mn^2+^ ions were removed, CML/Glutamine and MG-H1/Tyrosine adducts at the following specified locations were built using PyMOL (Schrödinger LLC, 2024) (RRID:SCR_000305) for the specified sites [H2B(K/CML/Q) 43, H4(K/CML/Q) 31, and H3(R/MG-H1/Y) 42] as follows:

Lys-to-Gln and Arg-to-Tyr substitutions were introduced using the PyMOL mutagenesis tool, whereas CML and MG-H1 adducts were built using the PyMOL builder. The rotamer of the lowest free energy was selected after applying the clean command to each built rotamer, including atoms within 6 Å. The protonation state of CML at physiological pH was determined as described previously (Belinskaia et al., 2024), residue name was changed to CML. Regarding the protonation state of the MG-H1 adduct, it is expected to be uncharged at physiological pH, as the pKa of the hydroimidazolone group of MG-H1 has been estimated to be 4.58, with a calculated value of 3.58 (Wang et al., 2012). The residue name was changed to MGH.

Given that there are two histones of the same type per NCP (corresponding to two chains in the PDB file), any change was introduced for both histones. Additionally, two systems were prepared, each containing either all mutations or all glycation adducts (Table 1).

**TABLE 1:** Different built NCP systems and the corresponding amino-acid substitutions and glycation modifications.

NCP name	Histone type-modification (chains)
WT	Wild-type
GLY	H2BK43CML (D, H), H3R42MG-H1 (A, E), H4K31CML (B, F)
MUT	H2BK43GLN (D, H), H3R42TYR (A, E), H4K31GLN (B, F)
H2BK43CML	H2BK43CML (D, H)
H2BK43Q	H2BK43GLN (D, H)
H3R42MGH	H3R42MG-H1 (A, E)
H3R42Y	H3R42TYR (A, E)
H4K31CML	H4K31CML (B, F)
H4K31Q	H4K31GLN (B, F)

### 2.3 Simulation parameters

The systems were simulated using Amber14 and AmberTools15 (Case et al., 2015) (RRID: SCR_014230). The AMBER ff14SB force field (Maier et al., 2015) was used for proteins, combined with parmbsc1 corrections (Ivani et al., 2015) for DNA, and the TIP3P water model (Jorgensen et al., 1983). All systems were placed in a truncated octahedron simulation box with periodic boundary conditions set at least 14 Å away from the NCP atoms. Na^+^ and Cl^−^ ions were added to neutralize the charge and bring the ionic strength to 150mM using the LEaP module.

#### 2.3.1 Minimization, equilibration, and MD production

The solvated systems underwent a multi-step minimization and equilibration protocol. Each system was initially optimized with 10,000 cycles of energy minimization (100 steepest descent followed by 9,900 conjugate gradient), applying positional restraints of 100 kcal mol^-1^·Å^-2^ on the NCP atoms and disabling the SHAKE algorithm. The system was then heated from 100 to 310 K over 1 ns under constant volume (NVT ensemble). The temperature was subsequently maintained at 310 K using a Langevin thermostat with a collision frequency of 1.0 ps^-1^. Heating was followed by 1 ns equilibration under constant pressure (1 bar, NPT ensemble) using a Monte Carlo barostat, both with the same positional restraints. Subsequent equilibration stages under constant pressure gradually reduced the positional restraints on the NCP atoms from 100 to 10 kcal mol^-1^·Å^-2^ over 1 ns, after which an additional 10,000-cycle minimization (300 steepest descent and 9,700 conjugate gradient) was performed with 10 kcal mol^-1^·Å^-2^ restraints on the NCP backbone atoms only. The restraint strength was then progressively decreased to 1 and 0.1 kcal mol^-1^·Å^-2^ over successive 1 ns intervals. Finally, an unrestrained 1 ns equilibration was conducted. Gradual heating from 100 K to 310 K was used to allow the solvated nucleosome system to adjust progressively to the target temperature and to reduce the risk of abrupt structural perturbations before production MD. The additional minimization step with reduced backbone restraints was included to relax remaining unfavorable local contacts after initial restrained equilibration while preserving the overall nucleosome architecture. Translational and rotational centre-of-mass motions were removed every 1,000 steps. MD production runs were executed using pmemd.cuda (CUDA 7.5). All bonds involving hydrogen were constrained using SHAKE (ntc = 2, ntf = 2). For the 100 ns simulations, three independent replicate trajectories were generated for the WT system and for each single-site system (H2BK43CML, H2BK43Q, H3R42MGH, H3R42Y, H4K31CML, and H4K31Q) from the corresponding equilibrated structure, using a 2 fs time step and different random seeds for the Langevin thermostat. A cutoff of 8 Å was used for nonbonded interactions, and long-range electrostatic interactions were treated using the Particle Mesh Ewald (PME) method. Simulations were carried out under constant-pressure periodic boundary conditions (1 bar) using a Monte Carlo barostat.

For the initial 1 µs production trajectories, the first of the three 100 ns replicate trajectories for each system was selected and extended to 1 µs by running consecutive 100 ns segments because of cluster wall-time limits and the high computational cost of microsecond-scale simulations of full nucleosome systems. In addition, the GLY and MUT systems were each simulated for 1 µs using consecutive 100 ns segments initiated from the equilibrated structure. In all extended trajectories, continuation between segments was performed by reading coordinates and velocities from the previous restart file. To further assess reproducibility of the H3R42Y-associated DNA-end dynamics, the remaining two 100 ns replicates of WT and H3R42Y were subsequently also extended to 1 µs. The 100 ns triplicate simulations were used to assess reproducibility of local residue-level trends, whereas the 1 µs trajectories were used to examine longer-timescale structural behavior and are interpreted as structural observations rather than population-level statistical estimates.

To suppress potential fraying of terminal DNA base pairs during simulations, a harmonic distance restraint (force constant = 1.5 kcal mol^-1^·Å^-2^) was applied between the glycosidic nitrogen atoms (N1 of pyrimidines and N9 of purines) of the terminal base pairs, maintaining the base-pair geometry throughout the simulations.

### 2.4 Trajectory analysis for the 100 ns simulations

The properties of the MD trajectories, including temperature, density, and energy were extracted using process_mdout.perl code from the AmberTools and visualized with R.

For local hydrogen-bond and contact analyses of the 100 ns simulations, site-centered sub-trajectories were generated by extracting residues within 20 Å of each residue of interest, as defined from the starting structure in PyMOL.

#### 2.4.1 Root-mean-square deviations (RMSD)

The RMSDs of all systems were calculated with respect to the initial structure throughout 100 frames corresponding to 100 ns simulations using cpptraj, and then R and RStudio were used to calculate the average RMSD for replicates and visualize the data (Posit team, 2024; R Core Team, 2024) (R Project for Statistical Computing (RRID:SCR_001905), RStudio (RRID:SCR_000432)).

#### 2.4.2 Root-mean-square fluctuations (RMSF)

Cpptraj was used to calculate the RMSF for each residue using only heavy atoms (excluding hydrogens) over 100 frames spanning 100 ns. R and RStudio were used to calculate the average RMSF for replicates and visualize the data (Posit team, 2024; R Core Team, 2024).

#### 2.4.3 Hydrogen bond and salt bridges analysis

Hydrogen-bond and salt-bridge analyses were performed on these sub-trajectories using 1,000 frames spanning 100 ns. Only intermolecular hydrogen bonds were considered.

The angle cutoff used for hydrogen-bond and salt-bridge analyses was 135° (default). For hydrogen bonds, the donor–acceptor distance cutoff was 3.5 Å, while for salt bridges, it was 5 Å.

The salt-bridge interactions were filtered using R taking into account the different donor/acceptor atoms mentioned in Table 2.

**TABLE 2:** Salt-bridge interactions donor/acceptor atoms.

Salt-bridge donor atoms	
Residue	Donor atoms
Arginine	NH1, NH2
Lysine	NZ
CML	N01

Salt-bridge acceptor atoms	
Residue	Acceptor atoms
DNA	OP2
Glutamic acid	OE2
Aspartic acid	OD2
CML	O03, O01

The fractions of all detected hydrogen bonds between each residue of interest and other residues were summed to obtain the total hydrogen-bond fraction for each simulation. The salt-bridge and hydrogen-bond output data were analyzed and visualized in MS Excel. The average was calculated using the three replicates of each system.

#### 2.4.4 Contact analysis

Native-contact analysis was performed on the same sub-trajectories using 1,000 frames and a 5.5 Å cutoff. Contact frequencies for identical atom pairs were first averaged across replicates, retaining only contacts detected in all replicates, and the summed contact fractions were then used as contact scores.

#### 2.4.5 Solvent-accessible surface area (SASA)

To analyze the accessibility of the residues of interest across different systems, SASA was calculated with AmberTools using the LCPO (Linear Combinations of Pairwise Overlaps) algorithm. SASA was calculated for each residue over 100 frames spanning 100 ns, after which the mean across frames was determined, with negative values set to zero. The resulting values were then averaged across the three replicates for each system.

### 2.5 Trajectory analysis for the 1 µs simulations

Long simulation analysis included 5,000 frames per simulation (a frame every 0.2 ns), hydrogen bonds and salt bridges, and RMSD and RMSF calculated over the same number of frames. RMSF for the double-stranded DNA was calculated as the average of the DNA base-pair RMSF scores.

Hydrogen bond fraction and salt-bridge fraction of double-stranded DNA were calculated as the sums of interactions detected for both nucleotides in a DNA base pair.

#### 2.5.1 H3-latch RIN analysis for the 1 µs simulations

Hydrogen bond residue interaction network (RIN) and contact RIN analyses were performed for the histone H3-latch using StructureViz, Cytoscape and UCSF Chimera 1.18 (Shannon et al., 2003; Pettersen et al., 2004; Morris et al., 2007) (Cytoscape (RRID:SCR_003032), UCSF Chimera (RRID:SCR_004097)). For hydrogen bonds RIN, hydrogen bonds occupancy was calculated with a discard-edge threshold of 0.1, and relaxing the H-bonds constraints by 0.5 Å. Regarding the contacts RIN, occupancy was calculated with a discard-edge threshold of 0.1, the Contact Parameters included finding atoms with VDW ≥ −0.4, contacts of pairs 4 or fewer bonds apart were ignored. Core DNA residues and their related edges were removed from the contact RINs for easier visualization. H3-latch RIN analysis was performed on the initial 1 µs trajectories used for the long-timescale comparison and was not extended to the additional WT and H3R42Y replicate trajectories.

### 2.6 Data analysis and visualization

The tidyverse, ggplot2, and dplyr R libraries were used for the analysis and data visualization. Processed simulation data were organized and plotted in Microsoft Excel, and final Figures were prepared in Microsoft PowerPoint.

UCSF Chimera 1.18 was used to measure angles, visualize MD trajectories and export snapshots and videos (Pettersen et al., 2004). PyMOL 3.0.3 was used to visualize and align the snapshots from the MD trajectories using super command.

The simulations and analyses with AmberTools were run on the IANVS high-performance computing cluster at Martin Luther University Halle-Wittenberg. The R analysis and Native contacts analysis using AmberTools23 (Case et al., 2023), which were performed on an HP Laptop 15s-fq5xxx.

## 3 Results

### 3.1 The 100 ns simulation results

For each histone type, both identical copies present in the nucleosome core particle (which are represented as separate chains in the structure) were analyzed independently. At each site of interest, the residue was present either in the wild-type form, as a glycated residue, or as a mutation/amino-acid exchange introduced to mimic glycation. Nucleosome stability was first confirmed by root-mean-square deviation (RMSD) analysis (Supplementary Figure S1). Subsequent analyses focused on local residue dynamics, including protein and DNA contacts, hydrogen bonding, salt-bridge interactions, residue flexibility (RMSF), and solvent-accessible surface area (SASA). An overview of the investigated sites is shown in Table 3.

**TABLE 3:** An overview of the investigated modifications in the nucleosome core particle (NCP) 5AV6.

Histone (chains)	Site of interest	Residue at the site of interest		
		Wild-type	Glycation	Mutation
H2B (D, H)	43	Lysine	CML	GLN
H3 (A, E)	42	Arginine	MG-H1	TYR
H4 (B, F)	31	Lysine	CML	GLN

#### 3.1.1 Histone H2B (LYS/CML/GLN)43

**FIGURE 2:**
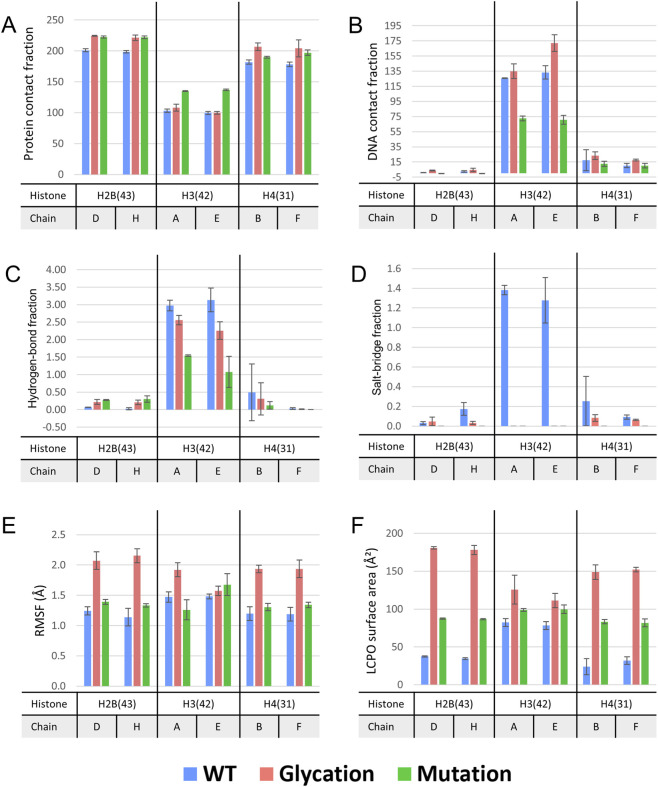
Local residue-level analysis from the 100 ns simulations. Bar plots showing mean values ±standard deviation for analyses of the residues of interest in the 100 ns simulations. **(A)** Protein contact fraction. **(B)** DNA contact fraction. **(C)** Hydrogen-bond fraction. **(D)** Salt-bridge fraction. **(E)** Root-mean-square fluctuation (RMSF). **(F)** Solvent-accessible surface area (SASA).

At position 43, LYS exhibited substantial protein contacts but minimal DNA interactions in both chains D and H. Despite the extent of protein contacts, hydrogen bonding and salt-bridge formation were infrequent. Substitution with CML or GLN resulted in an increase in protein contact frequency of approximately 11%–12% relative to LYS. DNA interactions remained low for all variants, with no detectable DNA contact observed for GLN. Hydrogen bond fractions were slightly higher for CML and GLN than for LYS (0.2–0.3) but remained low overall. Salt bridges were rarely observed for CML and absent for GLN, consistent with the lack of a charged side chain (Figures 2A–D; Supplementary Tables S1–S4).

Residue flexibility increased upon substitution of LYS with CML or GLN. CML displayed the largest increase in RMSF, with values elevated by 67% and 89% in chains D and H, respectively. GLN exhibited smaller but consistent increases of 12% and 17% relative to LYS (Figure 2E; Supplementary Tables S1–S4).

Solvent-accessible surface area (SASA) analysis revealed a pronounced increase in solvent exposure for both CML and GLN compared with LYS. CML showed increases of 384% and 418% in chains D and H, respectively, while GLN exhibited increases of 134% and 152% (Figure 2F; Supplementary Tables S1–S4).

#### 3.1.2 Histone H3 (ARG/MG-H1/TYR)42

At position 42, ARG engaged extensively with both protein and DNA in chains A and E, with average protein contact scores of approximately 100–103 and DNA contact scores of approximately 126–133. Conversion to MG-H1 increased DNA contacts by ∼7% in chain A and ∼29% in chain E, while protein contacts were unchanged in chain E and modestly increased (∼5%) in chain A. Substitution with TYR markedly increased protein contacts by 31% and 38% in chains A and E, respectively, but substantially reduced DNA contacts by approximately 42%–47%. ARG formed stable hydrogen bonds (fraction ∼3) and salt bridges (fractions of 1.38 and 1.28 in chains A and E, respectively). Hydrogen bonding decreased for MG-H1 by ∼14% and ∼28% in chains A and E, respectively, and was further reduced for TYR by ∼65% and ∼76%. Salt bridges were absent for MG-H1 and TYR (Figures 2A–D; Supplementary Tables S1–S4).

RMSF analysis indicated that MG-H1 exhibited increased flexibility relative to ARG, with RMSF increases of ∼31% in chain A and ∼6% in chain E. TYR showed chain-dependent behavior, with a 14% decrease in flexibility in chain A but a 14% increase in chain E (Figure 2E; Supplementary Tables S1–S4).

Within the H3R42 comparison, ARG displayed lower SASA values than its MG-H1 and TYR variants (∼82 and ∼78 Å^2^ in chains A and E, respectively). SASA increased by 53% and 42% for MG-H1 and by 20% and 28% for TYR in chains A and E, respectively (Figure 2F, Supplementary Tables S1–S4).

#### 3.1.3 Histone H4 (LYS/CML/GLN)31

Similar to H2B43, LYS31 in histone H4 showed high protein contact and limited DNA interaction in chains B and F, with notable variability in DNA contact in chain B. Hydrogen bonding and salt-bridge interactions were minimal. Glycation to CML increased protein contact by approximately 14% in both chains, while GLN substitution led to increases of 4% (chain B) and 11% (chain F). DNA contact increased for CML by 35% and 69% in chains B and F, respectively, although the absolute values remained low. In contrast, GLN reduced DNA contact by 27% in chain B and by 2% in chain F. Hydrogen bonds and salt bridges were largely absent for both CML and GLN (Figures 2A–D; Supplementary Tables S1–S4).

Flexibility increased for both modified residues relative to LYS. CML showed increases of 61% and 62% in chains B and F, respectively, while GLN exhibited smaller increases of 9% and 13% (Figure 2E; Supplementary Tables S1–S4).

SASA increased substantially for both substitutions. CML showed increases of 629% and 380% in chains B and F, respectively, while GLN exhibited increases of 308% and 158% (Figure 2F; Supplementary Tables S1–S4).

### 3.2 Summary of 100 ns simulations

**FIGURE 3:**
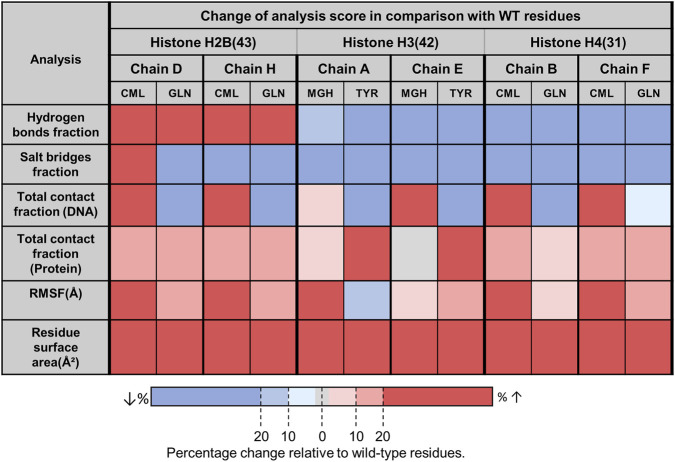
Relative effects of glycation and mutation compared with the corresponding wild-type residue. Percentage change in the analyzed properties for glycation and mutation relative to the corresponding wild-type residue. The figure provides a descriptive summary of the direction and relative magnitude of change across properties.

At positions H2B 43 and H4 31, LYS exhibited high protein contact and limited DNA interaction, both CML and GLN substitutions led to increased protein contact, flexibility, and solvent exposure relative to LYS, while DNA interactions, hydrogen bonding, and salt-bridge formation remained low. In contrast, H3R42 exhibited extensive interactions with both protein and DNA. At this position, TYR substitution shifted interactions toward increased protein contact and reduced DNA contact, whereas MG-H1 primarily enhanced DNA interactions without substantially altering protein contacts. Flexibility changes at H3R42 were residue- and chain-dependent, with MG-H1 generally increasing RMSF and TYR displaying divergent behavior between chains (Figures 2, 3; Supplementary Tables S1–S4).

### 3.3 The 1 µs simulation results

#### 3.3.1 H3R42 may contribute to double-stranded DNA (dsDNA) stabilization at the entry and exit sites

**FIGURE 4:**
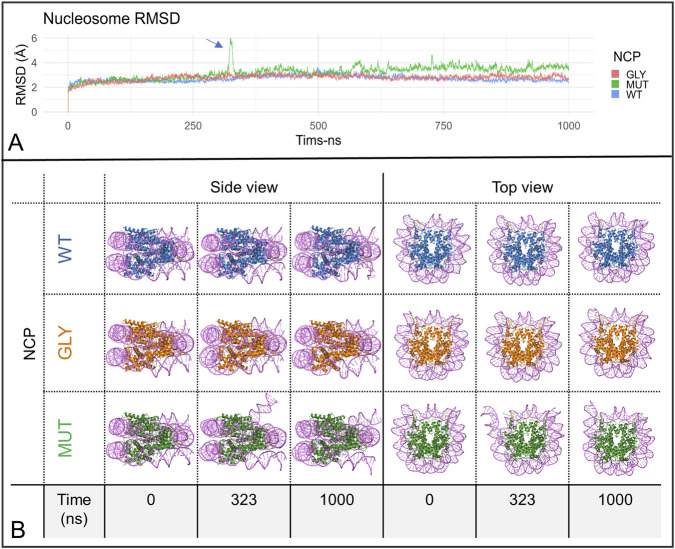
Global nucleosome dynamics in the 1 µs simulations. **(A)** NCP RMSD values during the 1 µs simulations. **(B)** Representative NCP snapshots from the 1 µs simulations shown in side and top views, including the first frame, a frame at 323 ns showing DNA breathing in the MUT system, and the last frame. Histone H3 residue 42 is highlighted in yellow.

**FIGURE 5:**
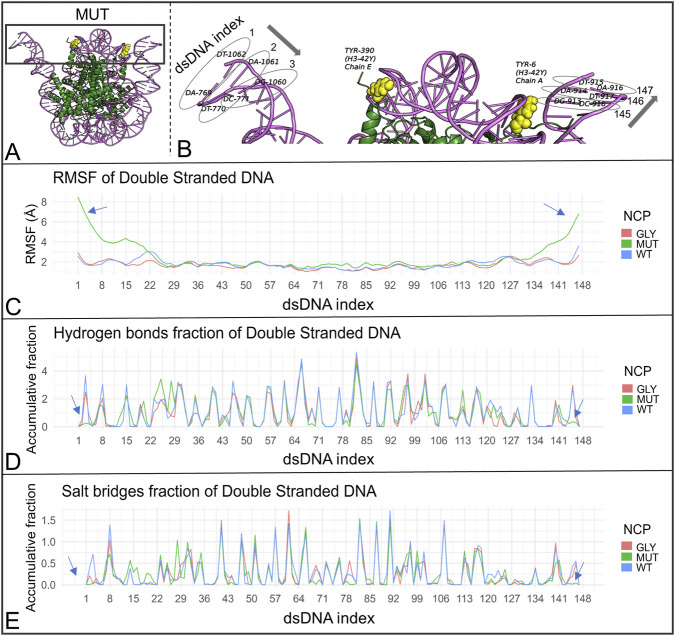
DNA breathing and DNA-end destabilization in the 1 µs simulations. **(A)** Snapshot of the MUT NCP at 323 ns showing DNA breathing near the H3R42Y substitution in chain E. **(B)** Introduced mutations highlighted in yellow together with the indexing of the double-stranded DNA. **(C–E)** Comparison of the WT, GLY, and MUT systems showing **(C)** RMSF, **(D)** hydrogen-bond fraction, and **(E)** salt-bridge fraction for the double-stranded DNA.

**FIGURE 6:**
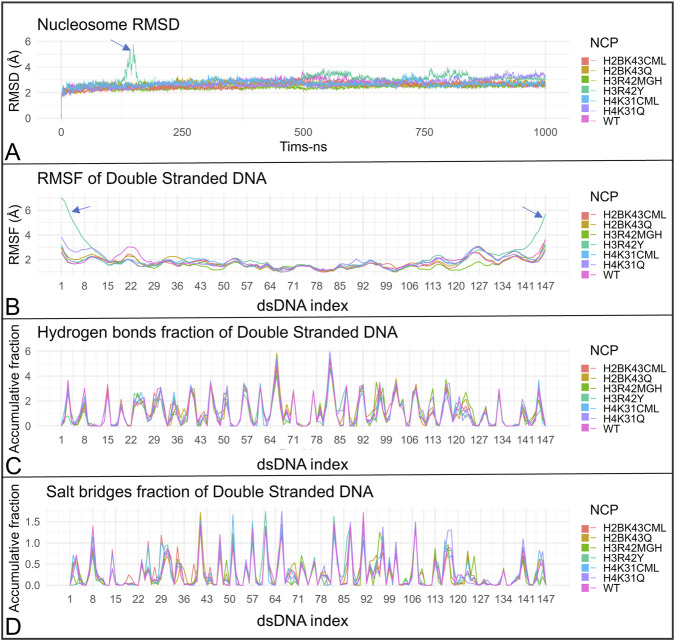
Effects of single-site modifications in the 1 µs simulations. Results from the 1 µs simulations of nucleosomes containing a single site-specific modification or substitution. **(A)** NCP RMSD values. **(B–D)** RMSF, hydrogen-bond fraction, and salt-bridge fraction, respectively, for the double-stranded DNA.

Three nucleosome core particle (NCP) systems were subjected to 1 µs MD simulation production runs: one unmodified, one with all glycated residues, and one with all mutations at the sites of interest (WT, GLY, and MUT). The objective was to assess whether these modifications exert global effects on NCP dynamics. The root-mean-square deviation (RMSD) scores across simulations indicated stability for both the wild-type and glycated nucleosomes. However, the nucleosome with mutations exhibited a notable increase in RMSD at approximately 322–328 ns, reaching a peak score of approximately 6 Å, followed by a slight but continuous increase compared to the other systems, ranging between approximately 3.5 Å and 4.5 Å, commencing at approximately 575 ns (Figure 4A). Visualization of the trajectories revealed DNA breathing at the entry/exit region of the dsDNA (Figures 4B, 5A; Supplementary Video S1). Here, ‘DNA breathing’ refers to transient entry/exit opening motions accompanied by increased end flexibility and reduced end stabilization, without implying sustained large-scale DNA unwrapping. The breathing was asymmetric, occurring at the DNA end proximal to the H3R42Y substitution in chain E (Figures 5A,B, Supplementary Figure S2). To further elucidate the nature of these dynamics, RMSF scores for the dsDNA were measured, revealing markedly higher flexibility at the DNA ends, with RMSF scores reaching approximately 8 Å (Figure 5C). Additionally, the fractions of hydrogen bonds and salt bridges between the dsDNA and the histone octamer were calculated, revealing a complete loss of these bonds for several DNA base pairs located at both ends of the dsDNA (Figures 5D,E). To test whether this behavior was linked specifically to H3R42Y rather than to the combined mutation, separate 1 µs MD simulations were conducted for NCPs with only one type of modification at a single site. The modification was applied to histones of the same type, resulting in two similar modifications at the same site, but on different histone chains within a single NCP. A similar RMSD peak was observed only in the nucleosome containing the TYR substitution at H3R42, detected between 135 ns and 155 ns (Figure 6A; Supplementary Video S2). There was also a total loss or a marked decrease in hydrogen bonding and salt-bridge formation between the dsDNA and the histone octamer for the terminal DNA base pairs. RMSF scores for dsDNA were measured, showing much higher flexibility at the DNA ends, with RMSF values reaching approximately 7 Å for the NCP with the TYR mutation at site 42 of histone H3 (Figures 6B–D).

Spearman correlation analysis was conducted to assess the relationship between the RMSF scores of dsDNA base pairs and the fractions of hydrogen bonds or salt bridges detected between them and the histone octamer. The results indicated a low but significant negative correlation between the RMSF scores and the hydrogen bond scores (Supplementary Figure S3), whereas a weaker, statistically non-significant (except for the H3R42Y NCP) negative correlation was observed between RMSF and salt bridge scores (Supplementary Figure S4).

To determine whether the chain-specific behavior seen in the initial 1 µs H3R42Y trajectory was reproducible across independent long-timescale runs, the remaining two 100 ns replicates of WT and H3R42Y were also extended to 1 µs. The three WT trajectories showed closely similar nucleosomal DNA RMSD and dsDNA RMSF profiles and did not exhibit asymmetric DNA breathing (Supplementary Figure S7A,B). In contrast, the additional H3R42Y trajectories showed replicate-dependent behavior: one displayed a later rise in nucleosomal DNA RMSD during the final ∼50 ns, reaching approximately 7 Å, whereas the other remained comparatively close to WT (Supplementary Figure S7A). A representative snapshot from the late-rising H3R42Y replicate shows asymmetric opening at one DNA end proximal to chain A (Supplementary Figure S7E). Despite this heterogeneity, all H3R42Y trajectories showed elevated dsDNA-end flexibility relative to WT, although the more affected DNA end differed between replicates (Supplementary Figure S7B). Consistent with this, histone–DNA hydrogen-bond and salt-bridge fractions at the DNA ends were generally reduced in the H3R42Y trajectories relative to WT (Supplementary Figure S7C,D). Together, these additional trajectories indicate that H3R42Y is associated with a reproducible, but replicate-dependent, propensity for asymmetric destabilization of nucleosomal DNA ends rather than a single fixed breathing pattern.

#### 3.3.2 H3-latch hydrogen bonds and contact RIN

##### 3.3.2.1 Hydrogen bonds RIN

In all initial 1 µs simulations included in the H3-latch RIN analysis, R397 exhibited partial hydrogen bonding with at least one of the three DNA residues DA775.I, DT776.I, and DT1059.J. At the site of interest (H3R42; residue 390 in chain E, see Table 2), no hydrogen bonding was detected in the MUT-NCP, and only low occupancy (0.241) with DG1060.J was observed in the H3R42Y system (Supplementary Figure S5). This reduction was accompanied by loss of hydrogen bonding with DA1061.J at the DNA entry/exit site. In contrast, high hydrogen-bond occupancy between H3R42 and DG1060.J was observed in all other simulations, while interactions with DA1061.J remained low.

Notably, whenever hydrogen bonding was detected between H3R42 and DG1060.J, the same DNA residue simultaneously formed hydrogen bonds with T393 (corresponding to H3T45), with comparable occupancy. Substitution of H3R42 with tyrosine resulted in loss of hydrogen bonding at both position 42 and T393, consistent with cooperative stabilization of DG1060.J within the H3-latch network.

Additionally, DT774.I formed occasional hydrogen bonds with either Y389 or HIE387, or both, across several NCP systems, independent of the presence of tyrosine at residue 390.

##### 3.3.2.2 Contacts RIN

In all initial 1 µs simulations included in the contact RIN analysis, excluding the one with the tyrosine mutant at H3R42, a strong DNA contact was observed, particularly with DT1059, DG1060, and DA1061 of chain J. In contrast, a markedly lower contact occupancy was observed in the MUT and H3R42Y simulations (Supplementary Figure S6).

Main H3-latch residues that directly interact with terminal DNA are listed in Table 4.

**TABLE 4:** Corresponding index numbers of H3-latch residues that directly interact with terminal DNA.

Amino acid	Index number in the simulation files	Corresponding index number in histone H3
HIE	H387	H39
ARG	R388	R40
TYR	Y389	Y41
ARG	**R390**	**R42**
THR	T393	T45
ARG	R397	R49

Bold values indicate the H3R42 glycation site.

## 4 Discussion

This study presents an *in silico* evaluation of site-directed mutagenesis as a strategy to model histone glycation, focusing on residue-specific effects and their impact on nucleosome stability. By combining multiple 100 ns simulations with extended 1 µs trajectories, we distinguish local perturbations from longer-timescale consequences for nucleosome dynamics and DNA engagement within the histone core.

Short (100 ns) simulations were used to assess mutation-based glycation mimics by comparing the direction and relative magnitude of changes induced by glycation and mutation relative to the wild type across multiple local structural properties. Mimic fidelity was evaluated from the overall pattern across properties, while taking into account the absolute baseline values of the underlying measurements, since opposite-direction percentage changes can be misleading when wild-type values are very low and the underlying absolute differences are small.

At H2B K43 and H4 K31, both CML glycation and GLN substitution induced similar directional changes relative to lysine, including increased flexibility, solvent exposure, and modestly elevated protein contacts, while DNA interactions remained low. Although the magnitude of these effects was greater for CML, the conserved trends indicate that glutamine may serve as a limited qualitative proxy for lysine-derived CML at these sites.

Although glutamine behaved as a limited qualitative proxy for CML at the lysine sites examined here, it has also been widely used to mimic acetylation (Iwasaki et al., 2011; Gorsky et al., 2016; Reda et al., 2024). This raises the possibility that, at some positions, CML itself may reproduce selected acetylation-like structural effects, an idea worth testing experimentally, given the long half-life of histones and the stability of CML (Commerford et al., 1982).

In contrast, tyrosine substitution did not reproduce the effects of MG-H1 glycation at H3R42. Whereas MG-H1 largely preserved protein contacts and enhanced DNA interactions, the tyrosine mutation caused a pronounced loss of DNA binding and a redistribution toward protein contacts. Hydrogen bonding and salt-bridge interactions were strongly reduced for TYR relative to both arginine and MG-H1, demonstrating opposing directionality and marked local divergence. Together, these results indicate that tyrosine is not a suitable local structural proxy for MG-H1 under the conditions tested here. This outcome also highlights that the Tyr substitution should be regarded as an empirical and chemically simplified surrogate rather than a chemically equivalent representation of MG-H1.

The 1 µs simulations revealed that an unsuitable mutation-based mimic at H3R42 was associated, in this model system, with altered nucleosome core particle dynamics. While the wild-type and glycated nucleosomes remained stable, systems containing H3R42Y showed a replicate-dependent propensity for increased nucleosomal DNA RMSD, elevated DNA-end flexibility, and asymmetric entry/exit DNA breathing motions. Additional 1 µs WT and H3R42Y replicates indicated that the side and timing of this behavior varied between H3R42Y trajectories, whereas WT trajectories remained consistent and did not display comparable breathing. The variation in onset time across the H3R42Y replicates and the MUT NCP suggests that longer simulations may reveal additional breathing events in the H3R42Y trajectory that remained comparatively close to WT over the 1 µs timescale sampled here. These observations suggest that H3R42Y does not impose one fixed structural pathway, but rather increases the likelihood of asymmetric DNA-end destabilization within the present simulation framework.

Analysis of DNA flexibility, interaction occupancies, and residue interaction networks identified disruption of the H3-latch as a possible mechanistic origin of the enhanced DNA-end dynamics. In wild-type and glycated nucleosomes, H3R42 participated in a cooperative interaction network with terminal DNA bases that remained intact upon MG-H1 glycation. In contrast, this network was disrupted in the tyrosine-mutant systems and was associated with asymmetric DNA breathing. Together, these observations provide a structural basis for how H3R42 modifications could influence nucleosome behaviors relevant to transcriptional regulation, consistent with prior work implicating H3R42 methylation in transcriptional regulation (Casadio et al., 2013).

These simulations are consistent with a role for H3R42 in stabilizing nucleosomal DNA at the entry and exit sites. They further suggest that perturbation of this residue alone may be sufficient, within the present simulations, to alter DNA end-wrapping dynamics and weaken stabilizing histone–DNA contacts at the entry/exit region. A limitation of this study is that simulations were performed on a nucleosome core particle (histone octamer + ∼147 bp core DNA), which by definition lacks linker DNA and linker histone H1, and the crystallographic construct contains truncated histone tails. Consequently, the observed effects primarily reflect intrinsic core histone–DNA interactions and may not fully capture modulation of entry/exit dynamics by histone-tail contacts or by constraints imposed by linker DNA and H1 in chromatosomes. In addition, only three glycation sites and two AGE chemistries were examined, so the findings should not be generalized to all histone glycation sites or mutation-based PTM mimics. The extended 1 µs simulations should also be interpreted as structural observations rather than population-level statistical estimates. Experimental validation using approaches such as nucleosome footprinting, biochemical stability assays, or Cryo-EM will be important to test whether the predicted effects occur *in vitro* or in cells.

## 5 Conclusion

This work provides a site-specific computational evaluation of mutation-based models of histone glycation in the nucleosome. At the two lysine sites examined, H2BK43 and H4K31, glutamine reproduced selected local trends induced by CML, including increased protein contact, flexibility, and solvent exposure, suggesting that it may serve as a limited qualitative proxy at these positions. In contrast, H3R42Y did not reproduce the local structural effects of MG-H1 and was associated with altered H3-latch interactions and a replicate-dependent propensity for asymmetric entry/exit DNA breathing in the simulations. Together, these findings suggest that mutation-based glycation models may be site-, chemistry-, and context-dependent, and they underscore the importance of structural validation before such models are used to draw conclusions about possible biological implications.

## Data Availability

The datasets presented in this study can be found in online repositories. The names of the repository/repositories and accession number(s) can be found below: https://doi.org/10.5281/zenodo.19677468, 10.5281/zenodo.19677468.
