# Supplementary material for: Site-specific evaluation of mutation-based mimics of histone glycation in the nucleosome
Source: Front Mol Biosci. 2026 May 18;13:1838713. doi: 10.3389/fmolb.2026.1838713 (PMC13222815; doi:10.3389/fmolb.2026.1838713)
Supplement: Supplementary file 3 [file DataSheet1.docx]

Supplementary Material

# Supplementary Figures and Tables

## Supplementary Figures


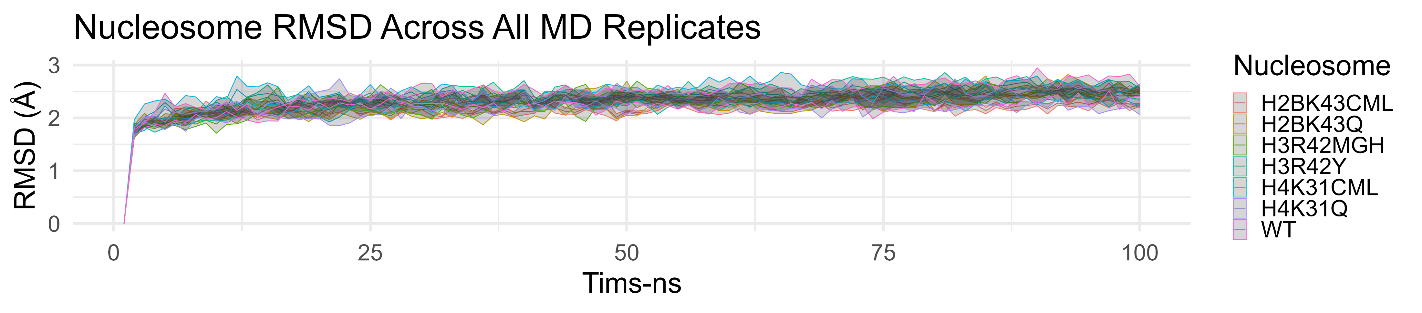


**Supplementary Figure S1.** Average RMSD scores for the 100ns simulations across three replicates.

**
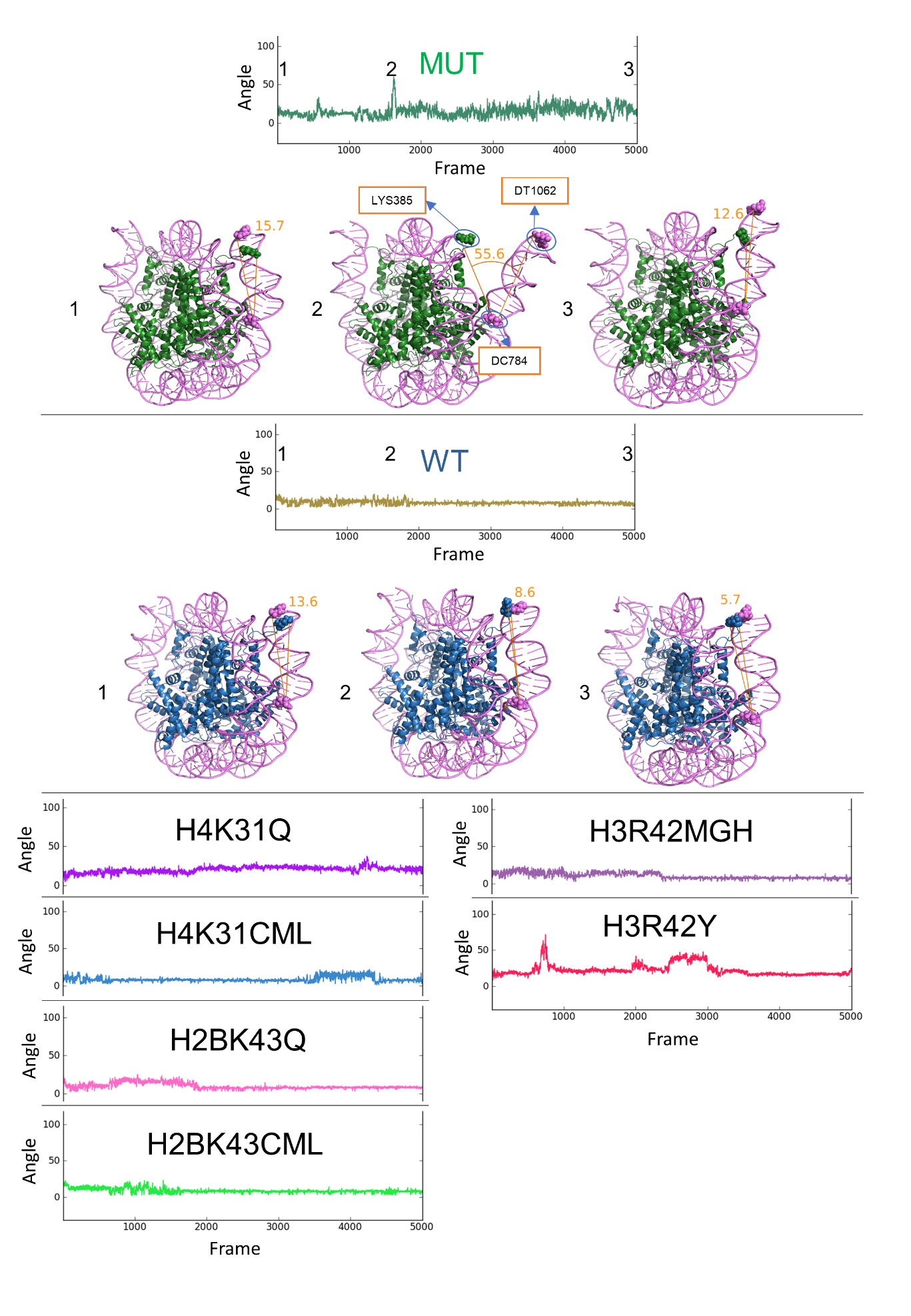
**

**Supplementary Figure S2.** Angle measurements for DNA entry/exit near residue 42 of Histone H3 (Chain E) across 5000 frames spanning 1µs trajectories. Angles were measured in UCSF Chimera 1.18 using the atoms :1062.J@OP2, :784.I@OP2, and :385.E@NZ. The NCP was visualized in PyMOL. The orange numbers indicate the measured angle values. LYS385, corresponding to H3K37, was used as the reference point because it lies within the histone core near the terminal DNA in the non-breathing state. The labeled snapshots correspond to 1 = 0 ns, 2 = 323 ns, and 3 = 1000 ns.


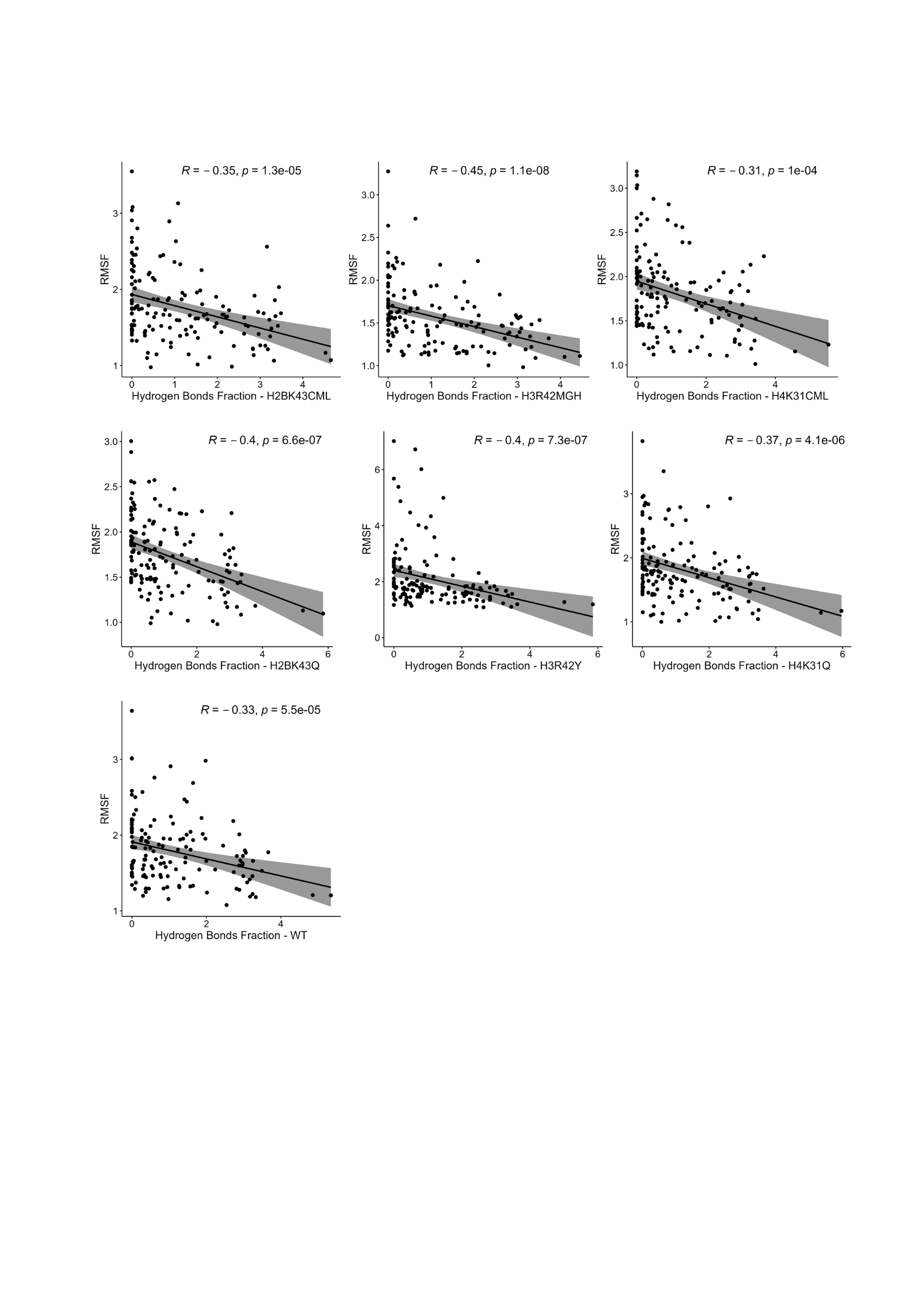


**Supplementary Figure S3.** Spearman correlation of RMSF vs dsDNA hydrogen bonds fraction for the 1µs trajectories.


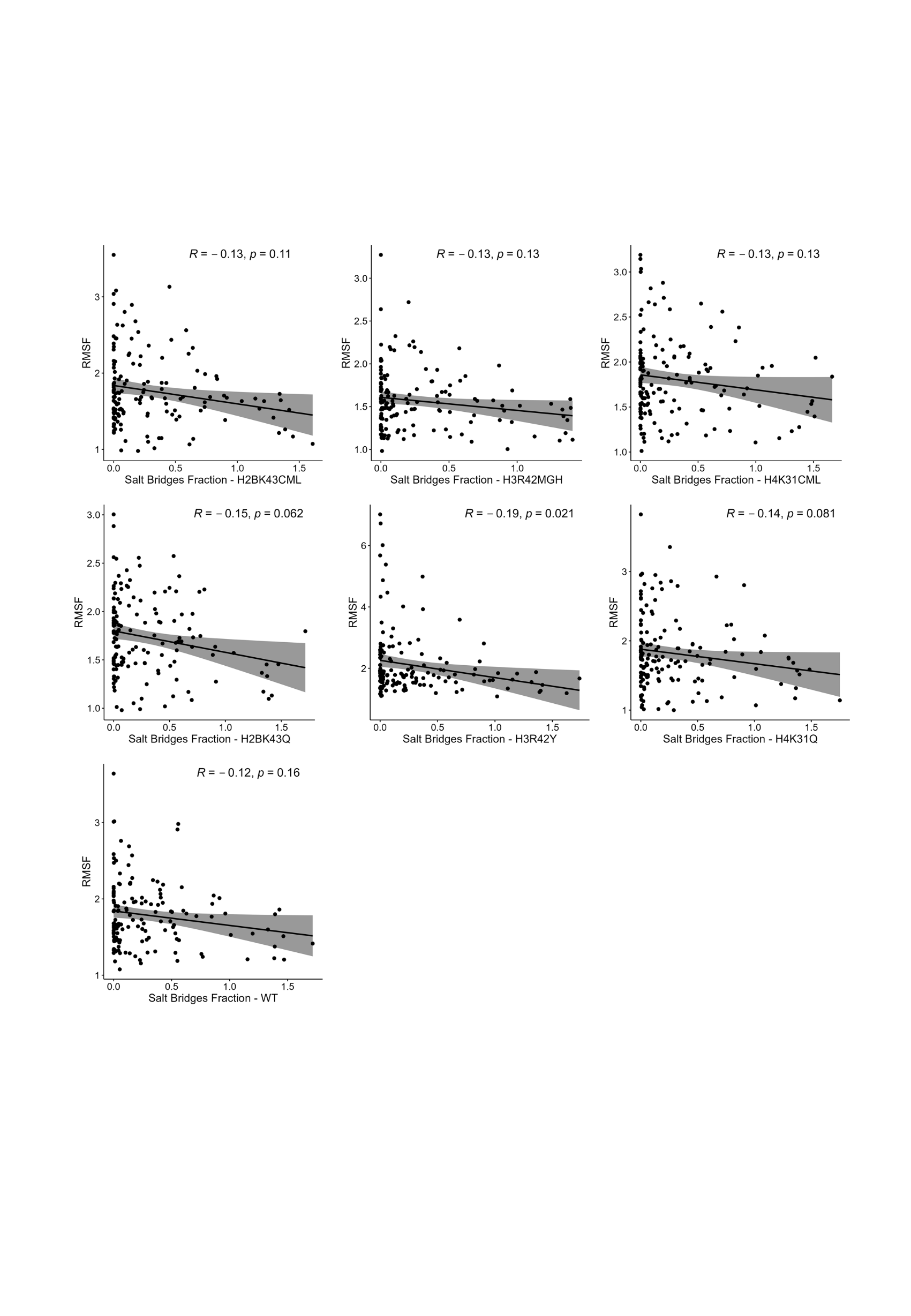


**Supplementary Figure S4.** Spearman correlation of RMSF vs dsDNA salt bridges fraction for the 1µs trajectories.


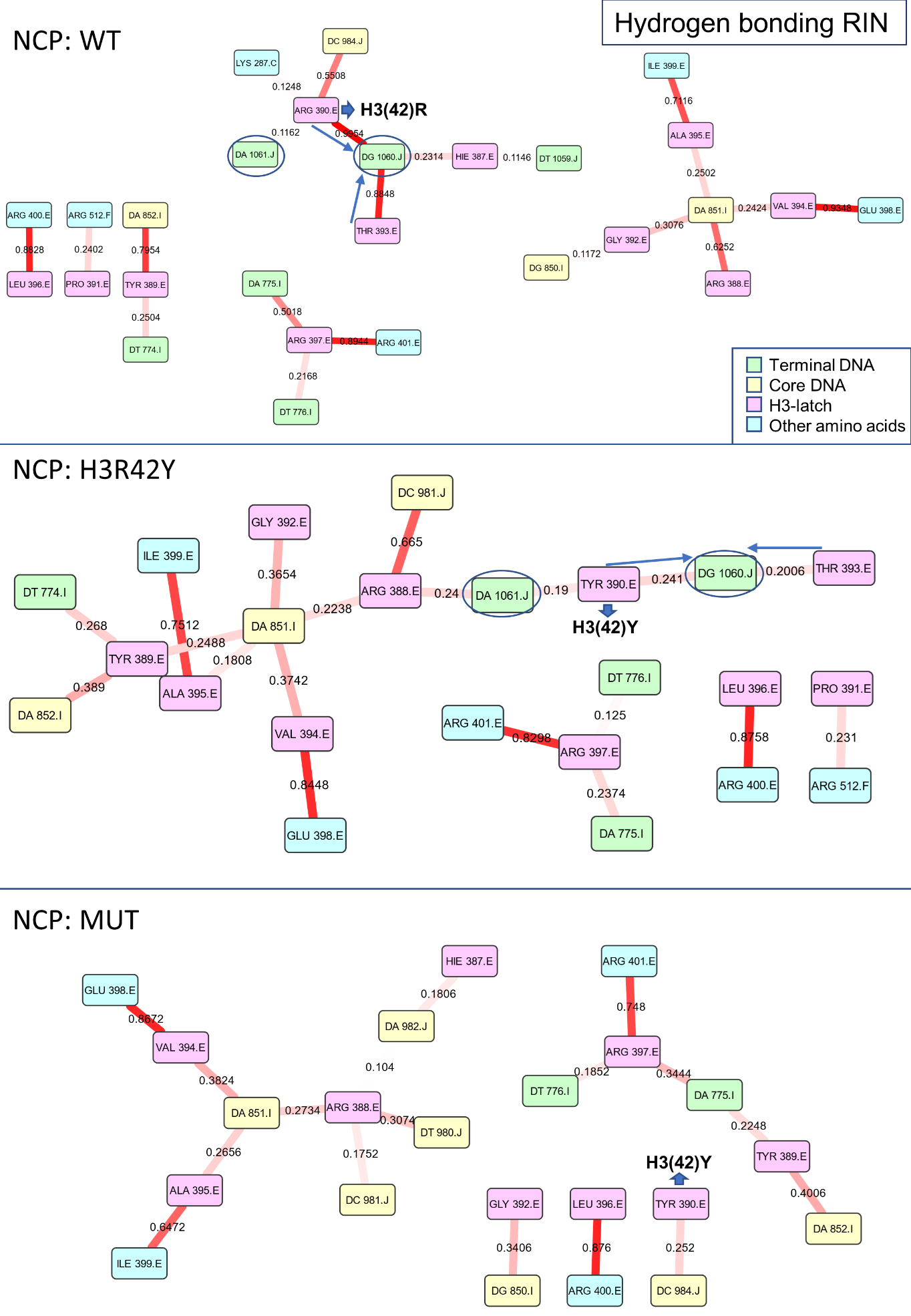


**Supplementary Figure S5.** Hydrogen bonding RIN for the H3-latch of 1 µs trajectories.


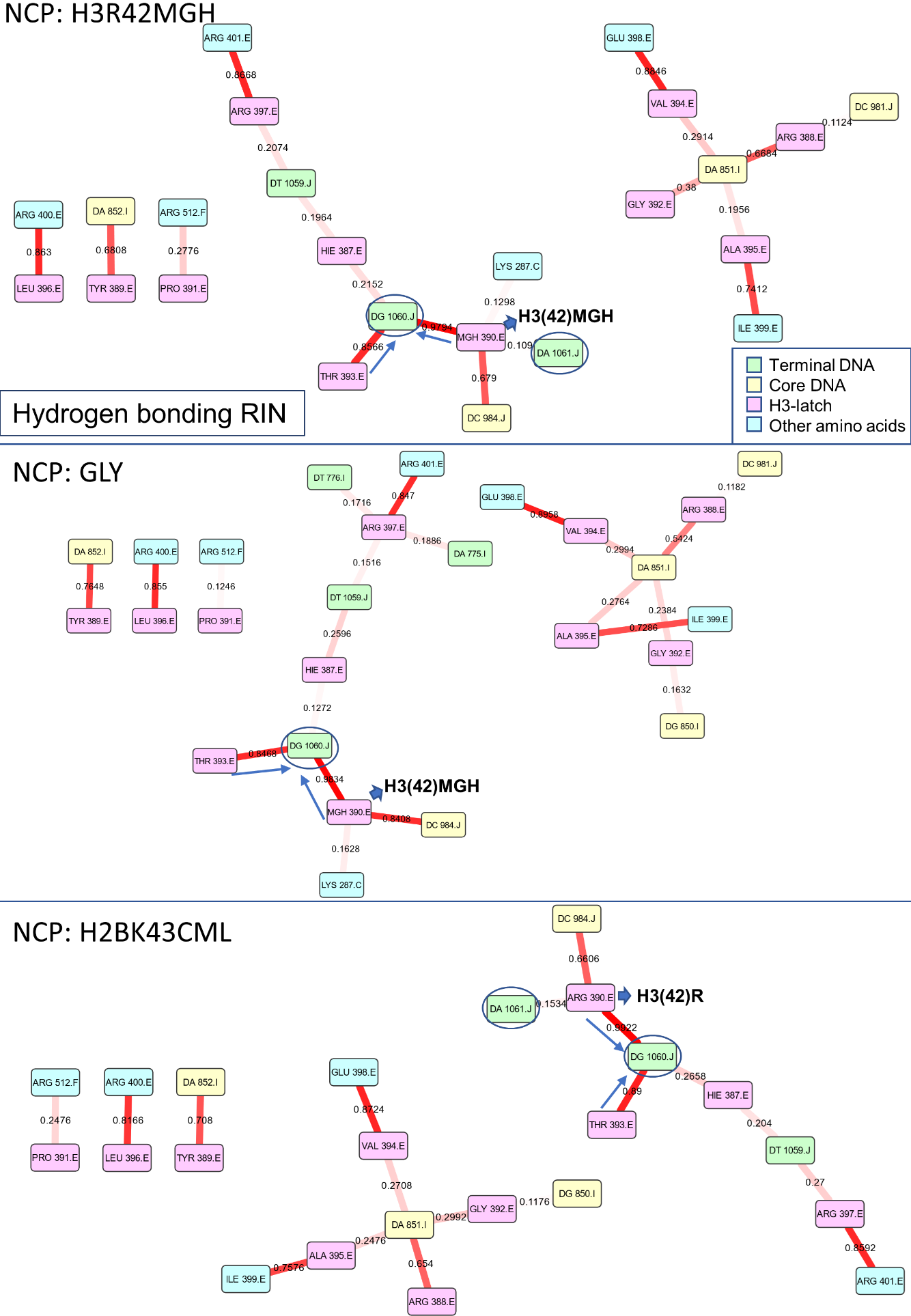


**Supplementary Figure S5.** Hydrogen bonding RIN for the H3-latch of 1 µs trajectories.


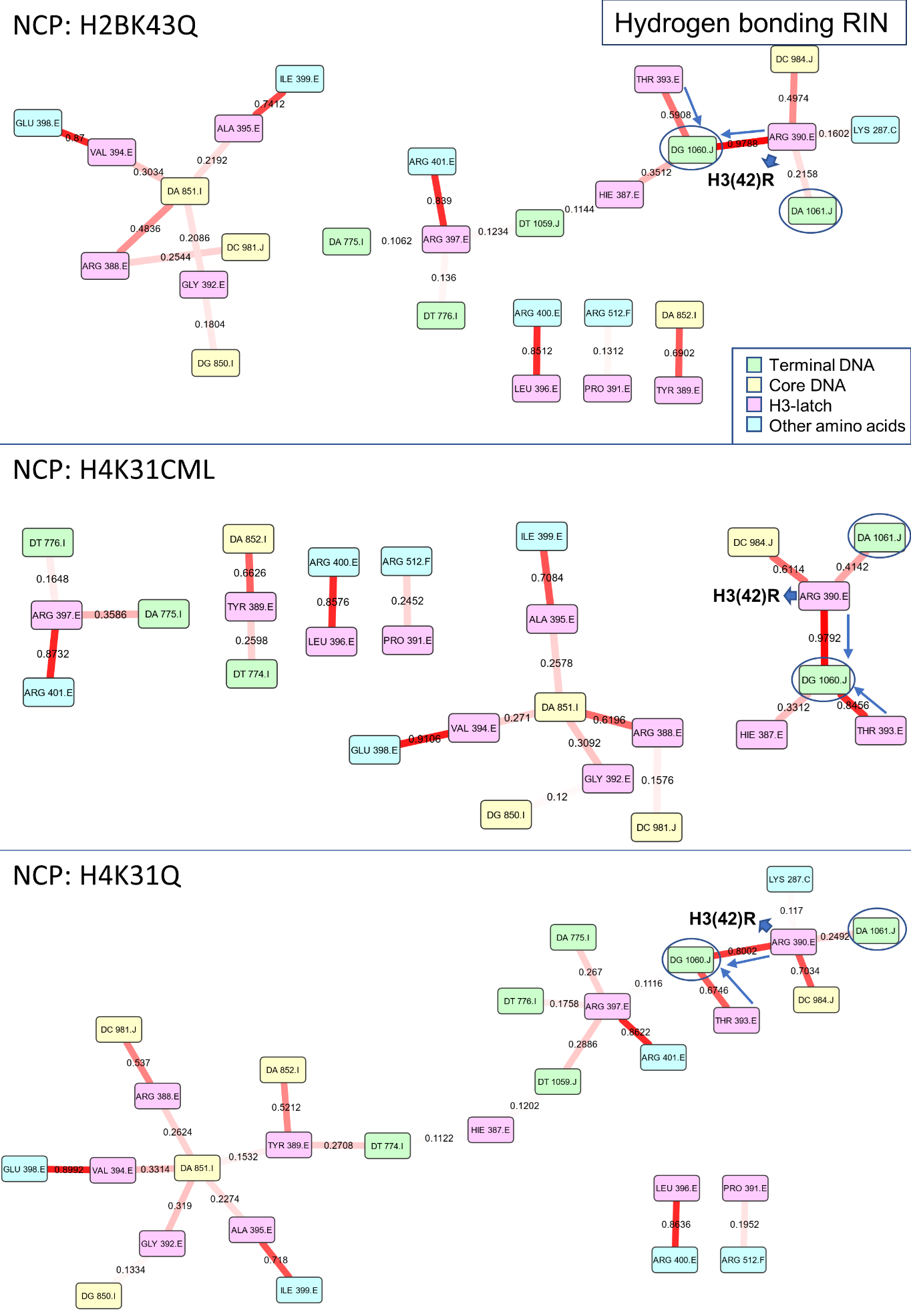


**Supplementary Figure S5.** Hydrogen bonding RIN for the H3-latch of 1 µs trajectories.


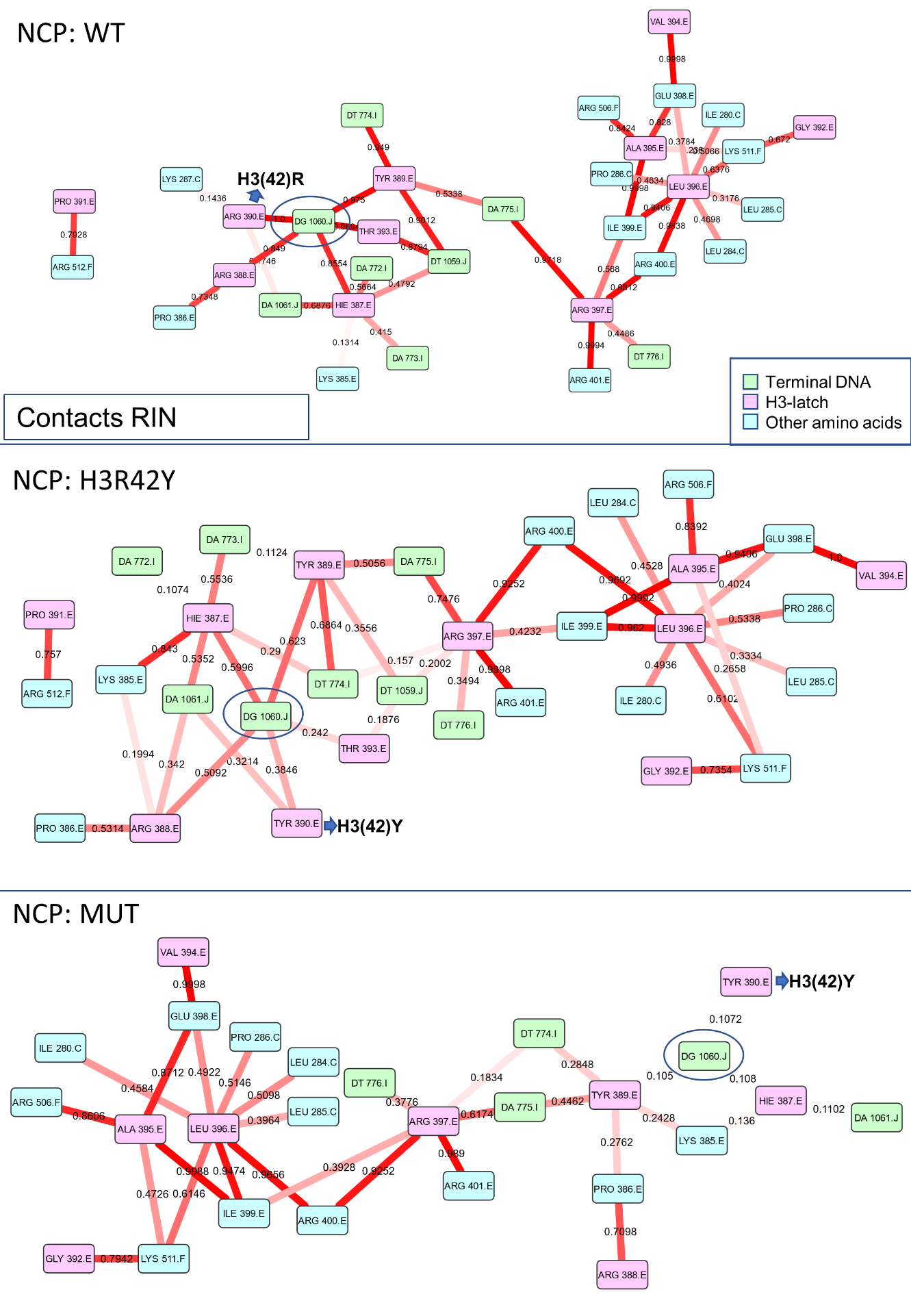


**Supplementary Figure S6.** Contacts RIN for the H3-latch of 1 µs trajectories.


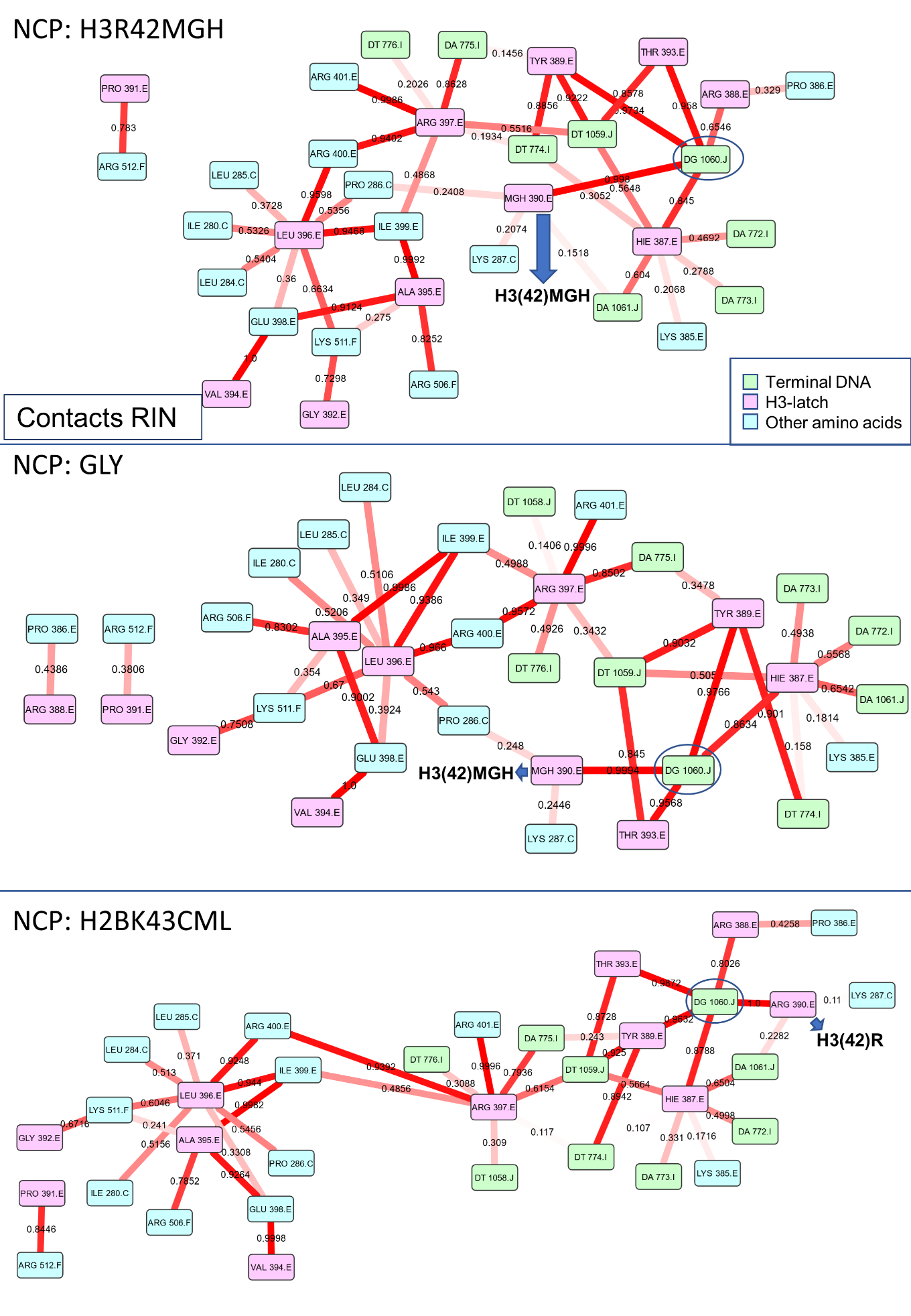


**Supplementary Figure S6.** Contacts RIN for the H3-latch of 1 µs trajectories.


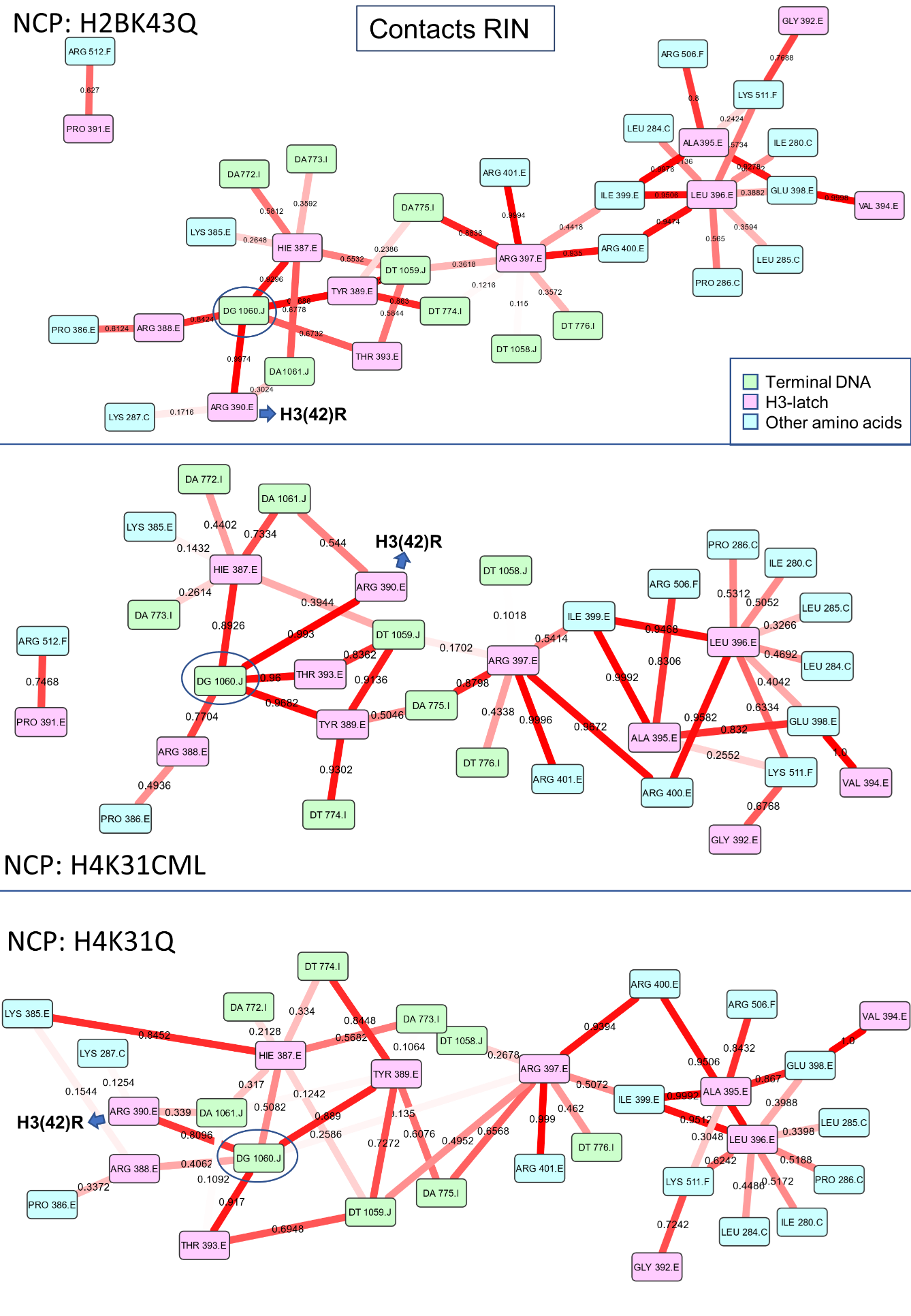


**Supplementary Figure S6.** Contacts RIN for the H3-latch of 1 µs trajectories.


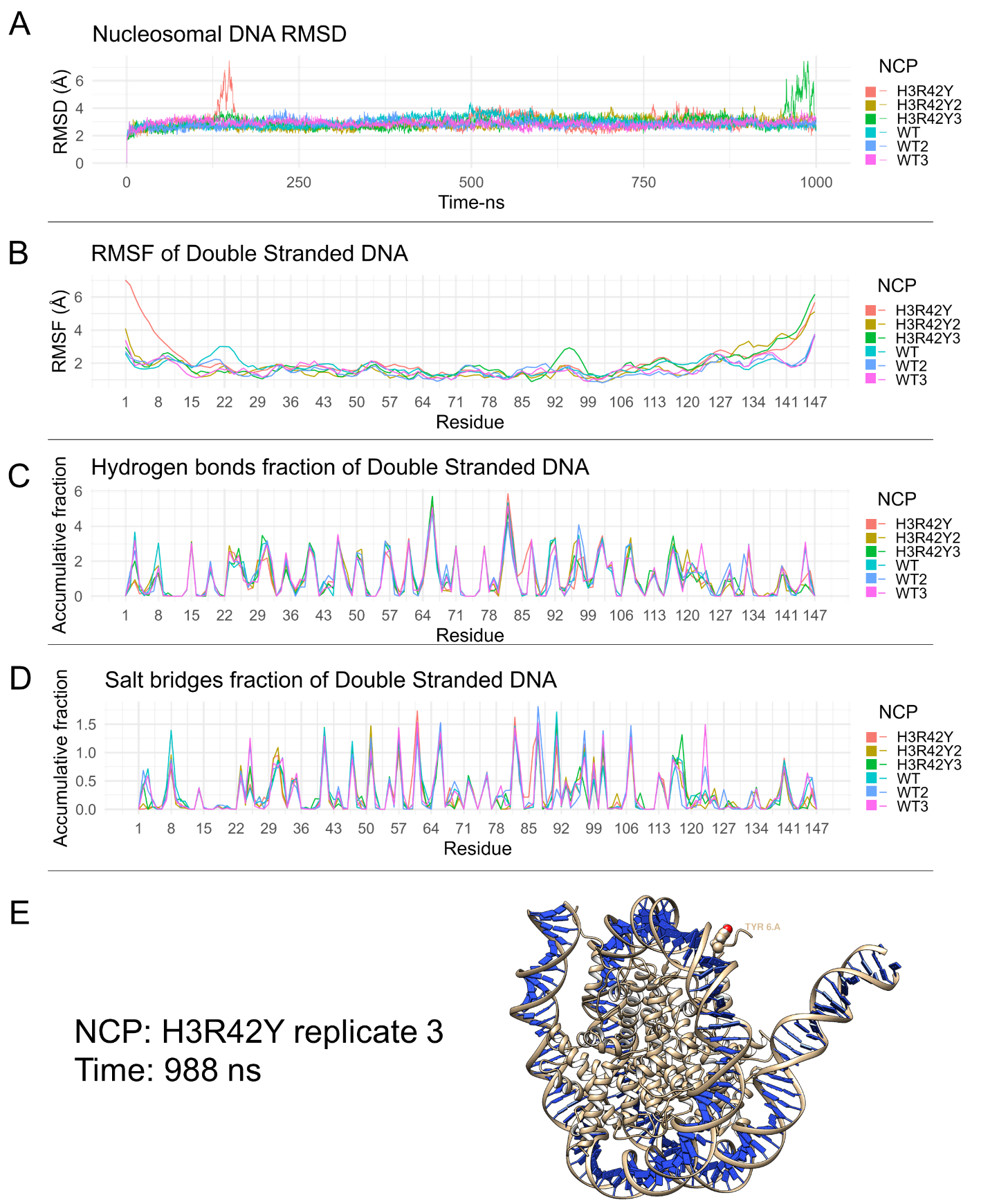


**Supplementary Figure S7.** Additional 1 µs replicate analysis of WT and H3R42Y nucleosomes.(A) Nucleosomal DNA RMSD over time for three WT and three H3R42Y trajectories.(B) Base pair-resolved dsDNA RMSF values.(C) Base pair-resolved cumulative hydrogen-bond fractions between dsDNA and the histone octamer.(D) Base pair-resolved cumulative salt-bridge fractions between dsDNA and the histone octamer.(E) Representative snapshot from the third H3R42Y 1 µs replicate at 988 ns, showing asymmetric DNA breathing.

## Supplementary Tables

**Supplementary Table S1 Average values for different residues.**

| Histone - Chain | Residue | Hydrogen bonds fraction | Salt bridges fraction | Total contact fraction (DNA) | Total contact fraction (Protein) | RMSF (Å) | Residue surface area (Å²) |
| --- | --- | --- | --- | --- | --- | --- | --- |
| Histone H2B - Chain D | **LYS** | 0.07 | 0.03 | 0.86 | 200.94 | 1.24 | 37.33 |
|  | **CML** | 0.22 | 0.05 | 3.66 | 224.33 | 2.07 | 180.7 |
|  | **GLN** | 0.28 | 0 | 0 | 222.49 | 1.39 | 87.52 |
| Histone H2B - Chain H | **LYS** | 0.02 | 0.17 | 2.62 | 198.64 | 1.14 | 34.37 |
|  | **CML** | 0.2 | 0.03 | 4.5 | 221.26 | 2.15 | 178.03 |
|  | **GLN** | 0.3 | 0 | 0 | 222.00 | 1.33 | 86.67 |
| Histone H3 - Chain A | **ARG** | 2.98 | 1.38 | 125.77 | 103.26 | 1.47 | 82.28 |
|  | **MGH** | 2.56 | 0.00 | 135.04 | 108.10 | 1.92 | 125.72 |
|  | **TYR** | 1.54 | 0.00 | 72.60 | 135.08 | 1.26 | 98.81 |
| Histone H3 - Chain E | **ARG** | 3.13 | 1.28 | 133.31 | 99.53 | 1.48 | 78.17 |
|  | **MGH** | 2.26 | 0.00 | 172.09 | 99.79 | 1.57 | 111.18 |
|  | **TYR** | 1.08 | 0.00 | 70.66 | 137.11 | 1.68 | 99.74 |
| Histone H4 - Chain B | **LYS** | 0.49 | 0.25 | 17.31 | 181.95 | 1.2 | 20.42 |
|  | **CML** | 0.31 | 0.08 | 23.37 | 206.77 | 1.93 | 148.8 |
|  | **GLN** | 0.12 | 0 | 12.63 | 189.95 | 1.31 | 83.26 |
| Histone H4 - Chain F | **LYS** | 0.03 | 0.09 | 10.34 | 178.32 | 1.19 | 31.7 |
|  | **CML** | 0.01 | 0.06 | 17.52 | 204.09 | 1.93 | 152.27 |
|  | **GLN** | 0 | 0 | 10.18 | 197.16 | 1.34 | 81.69 |

**Supplementary Table S2 Average values with the direction of change in comparison with the wild-type residue.**

| Histone - Chain | Residue | Hydrogen bonds fraction | Salt bridges fraction | Total contact fraction (DNA) | Total contact fraction (Protein) | RMSF (Å) | Residue surface area (Å²) |
| --- | --- | --- | --- | --- | --- | --- | --- |
| Histone H2B - Chain D | **LYS** | 0.07 | 0.03 | 0.86 | 200.94 | 1.24 | 37.33 |
|  | **CML** | 0.22↑ | 0.05↑ | 3.66↑ | 224.33↑ | 2.07↑ | 180.7↑ |
|  | **GLN** | 0.28↑ | 0↓ | 0↓ | 222.49↑ | 1.39↑ | 87.52↑ |
| Histone H2B - Chain H | **LYS** | 0.02 | 0.17 | 2.62 | 198.64 | 1.14 | 34.37 |
|  | **CML** | 0.2↑ | 0.03↓ | 4.5↑ | 221.26↑ | 2.15↑ | 178.03↑ |
|  | **GLN** | 0.3↑ | 0↓ | 0↓ | 222.00↑ | 1.33↑ | 86.67↑ |
| Histone H3 - Chain A | **ARG** | 2.98 | 1.38 | 125.77 | 103.26 | 1.47 | 82.28 |
|  | **MGH** | 2.56↓ | 0.00↓ | 135.04↑ | 108.10↑ | 1.92↑ | 125.72↑ |
|  | **TYR** | 1.54↓ | 0.00↓ | 72.60↓ | 135.08↑ | 1.26↓ | 98.81↑ |
| Histone H3 - Chain E | **ARG** | 3.13 | 1.28 | 133.31 | 99.53 | 1.48 | 78.17 |
|  | **MGH** | 2.26↓ | 0.00↓ | 172.09↑ | 99.79~ | 1.57↑ | 111.18↑ |
|  | **TYR** | 1.08↓ | 0.00↓ | 70.66↓ | 137.11↑ | 1.68↑ | 99.74↑ |
| Histone H4 - Chain B | **LYS** | 0.49 | 0.25 | 17.31 | 181.95 | 1.2 | 20.42 |
|  | **CML** | 0.31↓ | 0.08↓ | 23.37↑ | 206.77↑ | 1.93↑ | 148.8↑ |
|  | **GLN** | 0.12↓ | 0↓ | 12.63↓ | 189.95↑ | 1.31↑ | 83.26↑ |
| Histone H4 - Chain F | **LYS** | 0.03 | 0.09 | 10.34 | 178.32 | 1.19 | 31.7 |
|  | **CML** | 0.01↓ | 0.06↓ | 17.52↑ | 204.09↑ | 1.93↑ | 152.27↑ |
|  | **GLN** | 0↓ | 0↓ | 10.18~ | 197.16↑ | 1.34↑ | 81.69↑ |

**Supplementary Table S3 Change in percentage of the average values in comparison with the wild-type residue.**

| Histone - Chain | Residue | Hydrogen bonds fraction | Salt bridges fraction | Total contact fraction (DNA) | Total contact fraction (Protein) | RMSF (Å) | Residue surface area (Å²) |
| --- | --- | --- | --- | --- | --- | --- | --- |
| Histone H2B - Chain D | **LYS** | 0.07 | 0.03 | 0.86 | 200.94 | 1.24 | 37.33 |
|  | **CML** | 214% | 67% | 326% | 12% | 67% | 384% |
|  | **GLN** | 300% | -100% | -100% | 11% | 12% | 134% |
| Histone H2B - Chain H | **LYS** | 0.02 | 0.17 | 2.62 | 198.64 | 1.14 | 34.37 |
|  | **CML** | 900% | -82% | 72% | 11% | 89% | 418% |
|  | **GLN** | 1400% | -100% | -100% | 12% | 17% | 152% |
| Histone H3 - Chain A | **ARG** | 2.98 | 1.38 | 125.77 | 103.26 | 1.47 | 82.28 |
|  | **MGH** | -14% | -100% | 7% | 5% | 31% | 53% |
|  | **TYR** | -48% | -100% | -42% | 31% | -14% | 20% |
| Histone H3 - Chain E | **ARG** | 3.13 | 1.28 | 133.31 | 99.53 | 1.48 | 78.17 |
|  | **MGH** | -28% | -100% | 29% | 0% | 6% | 42% |
|  | **TYR** | -65% | -100% | -47% | 38% | 14% | 28% |
| Histone H4 - Chain B | **LYS** | 0.49 | 0.25 | 17.31 | 181.95 | 1.2 | 20.42 |
|  | **CML** | -37% | -68% | 35% | 14% | 61% | 629% |
|  | **GLN** | -76% | -100% | -27% | 4% | 9% | 308% |
| Histone H4 - Chain F | **LYS** | 0.03 | 0.09 | 10.34 | 178.32 | 1.19 | 31.7 |
|  | **CML** | -67% | -33% | 69% | 14% | 62% | 380% |
|  | **GLN** | -100% | -100% | -2% | 11% | 13% | 158% |

**Supplementary Table S4 Values of all 100ns simulation replicates.**

| Histone - Chain | Residue | Hydrogen bonds fraction | Salt bridges fraction | Total contact fraction (DNA) | Total contact fraction (Protein) | RMSF (Å) | Residue surface area (Å²) |
| --- | --- | --- | --- | --- | --- | --- | --- |
| Histone H2B - Chain D | **LYS1** | 0.07 | 0.05 | 0.8 | 198.5 | 1.3 | 38.1 |
|  | **LYS2** | 0.06 | 0.02 | 0.6 | 201.0 | 1.2 | 36.4 |
|  | **LYS3** | 0.08 | 0.03 | 1.1 | 203.3 | 1.2 | 37.5 |
|  | **CML1** | 0.28 | 0.10 | 3.4 | 225.1 | 2.1 | 180.0 |
|  | **CML2** | 0.23 | 0.02 | 3.3 | 223.5 | 2.3 | 182.6 |
|  | **CML3** | 0.15 | 0.03 | 4.3 | 224.3 | 1.9 | 179.5 |
|  | **GLN1** | 0.27 | 0.00 | 0.0 | 222.6 | 1.4 | 87.0 |
|  | **GLN2** | 0.27 | 0.00 | 0.0 | 220.9 | 1.3 | 88.4 |
|  | **GLN3** | 0.29 | 0.00 | 0.0 | 224.0 | 1.4 | 87.2 |
| Histone H2B - Chain H | **LYS1** | 0.02 | 0.21 | 1.4 | 200.9 | 1.0 | 35.7 |
|  | **LYS2** | 0.00 | 0.21 | 3.2 | 197.9 | 1.0 | 33.2 |
|  | **LYS3** | 0.06 | 0.10 | 3.3 | 197.1 | 1.3 | 34.6 |
|  | **CML1** | 0.17 | 0.02 | 6.8 | 226.4 | 2.1 | 171.6 |
|  | **CML2** | 0.17 | 0.05 | 4.1 | 218.3 | 2.0 | 179.0 |
|  | **CML3** | 0.28 | 0.02 | 2.6 | 219.0 | 2.3 | 183.5 |
|  | **GLN1** | 0.40 | 0.00 | 0.0 | 222.1 | 1.3 | 87.6 |
|  | **GLN2** | 0.22 | 0.00 | 0.0 | 224.2 | 1.3 | 86.6 |
|  | **GLN3** | 0.29 | 0.00 | 0.0 | 219.7 | 1.4 | 85.9 |
| Histone H3 - Chain A | **ARG1** | 3.15 | 1.33 | 126.0 | 106.4 | 1.6 | 76.2 |
|  | **ARG2** | 2.86 | 1.38 | 125.9 | 101.9 | 1.5 | 84.9 |
|  | **ARG3** | 2.93 | 1.43 | 125.4 | 101.5 | 1.4 | 85.8 |
|  | **MGH1** | 2.52 | 0.00 | 124.9 | 101.7 | 1.8 | 146.7 |
|  | **MGH2** | 2.45 | 0.00 | 136.1 | 110.7 | 2.1 | 121.3 |
|  | **MGH3** | 2.71 | 0.00 | 144.2 | 111.8 | 1.9 | 109.2 |
|  | **TYR1** | 1.57 | 0.00 | 76.2 | 135.3 | 1.2 | 97.0 |
|  | **TYR2** | 1.52 | 0.00 | 69.9 | 134.1 | 1.5 | 100.6 |
|  | **TYR3** | 1.53 | 0.00 | 71.8 | 135.8 | 1.1 | 98.8 |
| Histone H3 - Chain E | **ARG1** | 3.51 | 1.05 | 127.5 | 102.4 | 1.5 | 82.8 |
|  | **ARG2** | 3.03 | 1.51 | 129.0 | 98.5 | 1.4 | 78.8 |
|  | **ARG3** | 2.86 | 1.27 | 143.5 | 97.7 | 1.5 | 72.8 |
|  | **MGH1** | 2.41 | 0.00 | 160.9 | 102.2 | 1.5 | 116.3 |
|  | **MGH2** | 2.39 | 0.00 | 182.7 | 99.8 | 1.5 | 100.2 |
|  | **MGH3** | 1.97 | 0.00 | 172.7 | 97.4 | 1.7 | 116.9 |
|  | **TYR1** | 0.57 | 0.00 | 76.7 | 138.3 | 1.9 | 96.1 |
|  | **TYR2** | 1.36 | 0.00 | 69.9 | 135.6 | 1.4 | 96.9 |
|  | **TYR3** | 1.30 | 0.00 | 65.3 | 137.4 | 1.7 | 106.2 |
| Histone H4 - Chain B | **LYS1** | 1.43 | 0.54 | 33.2 | 178.8 | 1.0 | 11.7 |
|  | **LYS2** | 0.00 | 0.12 | 8.2 | 185.4 | 1.3 | 31.9 |
|  | **LYS3** | 0.05 | 0.10 | 10.5 | 181.6 | 1.3 | 27.8 |
|  | **CML1** | 0.84 | 0.05 | 29.1 | 205.0 | 1.9 | 154.4 |
|  | **CML2** | 0.00 | 0.08 | 19.3 | 213.8 | 2.0 | 154.4 |
|  | **CML3** | 0.08 | 0.12 | 21.6 | 201.5 | 1.9 | 137.6 |
|  | **GLN1** | 0.00 | 0.00 | 8.7 | 188.6 | 1.2 | 86.6 |
|  | **GLN2** | 0.15 | 0.00 | 14.6 | 191.4 | 1.3 | 81.6 |
|  | **GLN3** | 0.21 | 0.00 | 14.6 | 189.8 | 1.4 | 81.6 |
| Histone H4 - Chain F | **LYS1** | 0.00 | 0.08 | 7.6 | 175.0 | 1.1 | 36.7 |
|  | **LYS2** | 0.05 | 0.12 | 13.4 | 177.5 | 1.2 | 32.6 |
|  | **LYS3** | 0.04 | 0.08 | 10.0 | 182.5 | 1.3 | 26.6 |
|  | **CML1** | 0.00 | 0.07 | 16.3 | 219.6 | 1.7 | 148.8 |
|  | **CML2** | 0.01 | 0.06 | 17.4 | 198.4 | 2.1 | 153.8 |
|  | **CML3** | 0.02 | 0.06 | 18.9 | 194.3 | 2.0 | 154.2 |
|  | **GLN1** | 0.00 | 0.00 | 7.4 | 192.4 | 1.3 | 85.9 |
|  | **GLN2** | 0.00 | 0.00 | 13.4 | 200.5 | 1.4 | 75.9 |
|  | **GLN3** | 0.00 | 0.00 | 9.7 | 198.5 | 1.3 | 83.3 |

## Supplementary Videos

**Supplementary Video S1**. Molecular dynamics trajectories from the 1 µs simulations of the WT, GLY, and MUT nucleosome core particles. Residue 390, corresponding to histone H3 residue 42, is highlighted.

**Supplementary Video S2**. Molecular dynamics trajectory from the 1 µs simulation of the H3R42Y nucleosome core particle. Residue 390, corresponding to histone H3 residue 42, is highlighted.
